# Letter to the editor according to the study of Schwarze *et al*.

**DOI:** 10.5935/1518-0557.20190040

**Published:** 2019

**Authors:** Marscha Sinnema

**Affiliations:** 1 Amsterdam University Medical Center, Amsterdam (VUMC), The Netherlands

I was very interested in reading the article of Schwarze *et al*. (2018) named 'DHEA use to improve likelihood of IVF/ICSI success in patients with diminished ovarian reserve: A systematic review and meta-analysis'. In the article they combined the results of 5 different studies and concluded that DHEA supplementation prior to IVF treatment has a positive effect on the pregnancy rate and frequency of abortion, though no effect on the mean number of retrieved oocytes was found. As I was reading the article closely, I discovered some errors that should be checked and corrected.

First of all, in the 'materials and methods' section, clinical pregnancy is defined as "the identification of at least one embryo displaying cardiac activity". In figure 2, showing the clinical pregnancy rate per initiated cycle, the number of patients in the control group of the Barad study (Barad *at al*., 2007) is listed as 18. However, when reading the article of Barad *et al.* (2007) the clinical pregnancy rate is only 11, while the number of patients with positive hCG is 18. This does not match the earlier described definition of clinical pregnancy.

Secondly, the study of Wiser *et al*. (2010) performed 2 study cycles of IVF treatment with a total of 33 patients. In figure 2, the results of the first treatment cycle are processed with an outcome of 4 and 2 clinical pregnancies in the treatment and control group respectively. However, figure 3, showing the abortion rate, contains the results after the second treatment cycle, containing more clinical pregnancies. Both figures should include the results of the same treatment cycle to be able to make an accurate comparison. Additionally, figure 4, containing the mean number of oocytes recovered, states that the SD of the treatment group is 2.6 and the SD of the control group is 1.7. However, in the original study (Wiser et al., 2010), those numbers are being described as the mean oocyte retrieval from 'the cycle prior to the stud y cycle'. After the second cycle of IVF treatment those numbers are respectively 3.2 and 3.5 in de treatment and control group with a total of 26 and 25 patients. These are the numbers that should be taken into account in the results.

At last, the study of Vlahos *et al.* (2015) listed 6 live births out of 8 clinical pregnancies in the control group. However, figure 3 states that the abortion rate in this group is 6/8, while this is only 2/8.

All together, these errors might affect the overall results and could influence the conclusion. Therefore the errors should be checked and there should be determined whether adjustment of the conclusion is necessary.
